# Self-Assembly of Insulin-Derived Chimeric Peptides into Two-Component Amyloid Fibrils: The Role of Coulombic Interactions

**DOI:** 10.1021/acs.jpcb.3c00976

**Published:** 2023-07-26

**Authors:** Mateusz Fortunka, Robert Dec, Wojciech Puławski, Marcin Guza, Wojciech Dzwolak

**Affiliations:** † Faculty of Chemistry, Biological and Chemical Research Centre, University of Warsaw, Pasteur Street 1, 02-093 Warsaw, Poland; ‡ Bioinformatics Laboratory, Mossakowski Medical Research Institute, Polish Academy of Sciences, Pawinski Street 5, 02-106 Warsaw, Poland

## Abstract

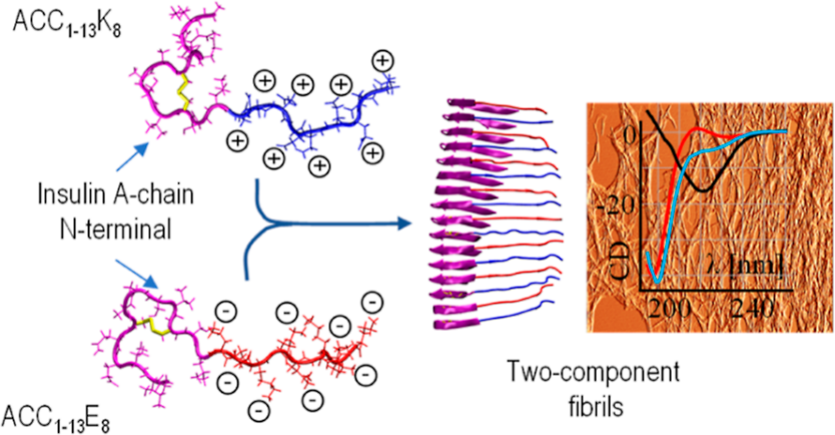

Canonical amyloid fibrils are composed of covalently identical polypeptide chains. Here, we employ kinetic assays, atomic force microscopy, infrared spectroscopy, circular dichroism, and molecular dynamics simulations to study fibrillization patterns of two chimeric peptides, ACC_1–13_E_8_ and ACC_1–13_K_8_, in which a potent amyloidogenic stretch derived from the N-terminal segment of the insulin A-chain (ACC_1–13_) is coupled to octaglutamate or octalysine segments, respectively. While large electric charges prevent aggregation of either peptide at neutral pH, stoichiometric mixing of ACC_1–13_E_8_ and ACC_1–13_K_8_ triggers rapid self-assembly of two-component fibrils driven by favorable Coulombic interactions. The low-symmetry nonpolar ACC_1–13_ pilot sequence is crucial in enforcing the fibrillar structure consisting of parallel β-sheets as the self-assembly of free poly-E and poly-K chains under similar conditions results in amorphous antiparallel β-sheets. Interestingly, ACC_1–13_E_8_ forms highly ordered fibrils also when paired with nonpolypeptide polycationic amines such as branched polyethylenimine, instead of ACC_1–13_K_8_. Such synthetic polycations are more effective in triggering the fibrillization of ACC_1–13_E_8_ than poly-K (or poly-E in the case of ACC_1–13_K_8_). The high conformational flexibility of these polyamines makes up for the apparent mismatch in periodicity of charged groups. The results are discussed in the context of mechanisms of heterogeneous disease-related amyloidogenesis.

## Introduction

Conversion of soluble polypeptides into insoluble amyloid fibrils is a complex, yet generic structural transition accessible to various proteins and synthetic peptides.^1–4^ Spontaneous formation of these assemblies in vivo is of great importance, as such fibrils (or early on-pathway intermediate aggregates) have been implicated in the etiology of several degenerative maladies including Alzheimer’s disease and type II diabetes mellitus.^5–7^ On the other hand, the remarkable thermodynamic^8,9^ and mechanical^10,11^ stability of amyloid fibrils has been long utilized by living organisms.^12–15^ Although amyloidal polymorphism is now a well-recognized phenomenon,^9,16,17^ there are common structural themes manifesting, in particular, on the level of individual protofilaments. These motifs are conducive to the saturation of favorable intermolecular interactions within the fibril and the simultaneous reduction of solvent exposure of nonpolar moieties. Satisfying these requirements is the sine qua non for amyloid fibrils to attain a level of stability rivaling that of the native state.^8^ Intuitively, saturation of interchain hydrogen bonds and short-distance van der Waals interactions between individual proteinaceous building blocks would be promoted, for example, when polypeptide chains are aligned in the form of tightly packed in-register parallel β-sheets—a structural motif often found in amyloid protofilaments.^16,17^ Hence, the typical spatial packing modes of protein backbones and side chains within amyloid fibrils harmonize with a quasi-translational symmetry of identical or nearly identical protein units, an important selection criterion in the case of low-symmetry building blocks.^18^ As a result, such a structural optimization makes a particular amyloid protofilament architecture compatible with a rather narrow set of polypeptide chains’ lengths, primary structures, and topologies. Thus, canonical amyloid fibrils tend to be rather homogeneous in terms of their chemical composition, although sporadic local modifications of the building block’s covalent structure can be accommodated (e.g., refs (19)–^22^). The situation is quite different when the amyloidogenic precursor with a large uncompensated electric charge requires binding to macromolecular counterions in order to form amyloid fibrils, as is, for example, observed for Tau protein interacting with heparin or poly-l-glutamic acid (poly-E).^23,24^ In this case, the charge-compensating polyions are expected to bind to the relatively disordered “fuzzy coat” region of Tau aggregates rather than to be incorporated within the amyloid core.^25^ In the realm of synthetic peptides, there are, however, several examples of strictly two-component amyloid-like fibrils where both components form the core structure (excellently reviewed in ref (26)). Formation of such fibrils, in which the self-assembly of two alternating building blocks is favored over one-component structures, is usually conditioned on either complementarity of electric charges (e.g., refs (27) and (28)), optimized 3D-packing through chiral modifications of one of the components (formation of so-called rippled β-sheet—e.g., refs (29) and (30)), or energetic preference for specific patterns of π–π stacking/dispersive interactions between both components.^31^ Gaining deeper insights into the principles of self-assembly of multicomponent amyloid aggregates is crucial for the effective design of functional fibrils (e.g., nanofibers with tunable optical and catalytic properties^26,32,33^). Equally important, however, is the biomedical context since in vivo cross-interactions of certain disease-associated amyloidogenic proteins may trigger the formation of heterogeneous fibrils (e.g., refs (34)–^36^). Novel insightful model systems to study these problems can be derived from the earlier identified highly amyloidogenic insulin fragment, ACC_1–13_, encompassing the A-chain’s disulfide-constrained N-terminal segment.^37–39^ The de novo fibrillization of ACC_1–13_ in aqueous solutions is remarkably fast, occurring without a detectable lag phase at both acidic and neutral pHs.^40^ On the other hand, two chimeric peptides, ACC_1–13_K_8_ and ACC_1–13_E_8_ created by extending the insulin’s amyloidogenic stretch by octalysine or octaglutamate fragments, respectively, do not aggregate at neutral pH due to strong Coulombic repulsion between monomers in solution. We have shown that fibrillization of ACC_1–13_K_8_ can be triggered by addition of ATP which becomes stoichiometrically incorporated within the amyloid.^41^ Likewise, fibrillization of ACC_1–13_E_8_ at a close-to-neutral pH is induced by multivalent metal cations.^42^ The initial motivation of this study was to explore the possibility of using the ACC_1–13_K_8_/ACC_1–13_E_8_ pair to synthesize two-component amyloid fibrils.

## Methods

### Samples

Peptides ACC_1–13_E_8_ (GIVEQCAASVCSLEEEEEEEE) and ACC_1–13_K_8_ (GIVEQCAASVCSLKKKKKKKK) were designed by extending the first 13 N-terminal residues of bovine insulin’s A-chain at the C-end by additional segments of 8 glutamate or 8 lysine residues, respectively. In both peptides, insulin’s original intrachain Cys6–Cys11 disulfide bond is retained, while the native Cys7 residue is substituted with Ala. ACC_1–13_E_8_, ACC_1–13_K_8_, ACC_1–13_, E_8_, as well as K_8_ peptides, all without N- or C-terminal modifications, were custom-synthesized by Pepscan (Lelystad, The Netherlands), typically at high purity exceeding 95%, and were delivered by the manufacturer as trifluoroacetic acid (TFA) salts. Poly-l-glutamic acid, poly-E (as a sodium salt, nominal molecular weight of 15–50 kDa); poly-l-lysine, poly-K (as a hydrobromide, nominal molecular weight of 30–70 kDa); polyallylamine, PAA (as a hydrochloride, nominal molecular weight of 50 kDa); branched polyethylenimine, PEI (nominal molecular weight of 25 kDa), and all other nonpeptidic chemicals were obtained from MilliporeSigma (Sigma-Aldrich). Due to the high glutamate or lysine contents, freeze-dried TFA salts (as provided by the manufacturer) of ACC_1–13_E_8_ and ACC_1–13_K_8_ dissolve in water easily and completely at a close-to-neutral pH. In this way, stock aqueous solutions of ACC_1–13_E_8_ and ACC_1–13_K, pH 7, typically at a 0.433 mM concentration, were obtained. Likewise, stock aqueous solutions of E_8_, K_8_, poly-E, poly-K, PAA, and PEI at similar corresponding weight concentrations and pH 7 were prepared using diluted HCl and NaOH for pH adjustment. Coaggregation was initiated by rapid mixing of appropriate volumes of stock aqueous solutions of negatively and positively charged components, all pH-preadjusted to 7, with the addition of proper volumes of H_2_O and stock solution of thioflavin T (ThT, 1 mM) to obtain samples with the compositions specified in figure captions. Unless stated otherwise, the stoichiometry of mixing was based on the desired mutual compensation (1:1) of negative and positive charges on both components and the assumed full ionization (or protonation) of carboxyl (or amine) groups at pH 7. The final concentration of ThT was 20 or 30 μM, as specified.

### Fibrillization Kinetics (Thioflavin T Fluorescence Assay)

ThT-fluorescence-based measurements (λ_ex._ 440 nm/λ_em._ 485 nm) of peptide fibrillization kinetics were carried out on a CLARIOstar plate reader from BMG LABTECH (Offenburg, Germany) using 96-well black microplates. Typically, each well was filled with a 150 μL portion of freshly prepared peptide solution containing ThT at a 20 or 30 μM concentration, as specified. Measurements were carried out at 37 °C and moderate agitation (300 rpm) for at least 24 h, as specified. Afterward, aggregate samples were collected from the plate and washed with portions of water in order to remove excess salts. Eluted pellets were subjected to atomic force microscopy (AFM) and Fourier transform infrared (FT-IR) spectroscopic measurements.

### Atomic Force Microscopy

Aggregate suspensions were collected from the plate at the end of the kinetic experiment and washed several times with water. Aqueous suspensions of aggregates were further diluted with water approximately five times. A small droplet (10 μL) of such a diluted suspension was swiftly deposited onto freshly cleaved mica and left to dry overnight. AFM tapping-mode measurements were carried out using a Nanoscope III atomic force microscope from Veeco Instruments (Plainview, NY, USA) and TAP300-Al sensors (res. frequency was 300 kHz) from BudgetSensors (Sofia, Bulgaria). We have also attempted to estimate the persistence lengths of various amyloid fibrils based on the AFM images. The methodological details are placed in the Supporting Information.

### Attenuated Total Reflectance FT-IR Measurements

Centrifuged samples of aggregates collected from the plate at the end of the kinetic experiment were washed several times with equal portions of water. Suspensions of fibrils were deposited and allowed to dry up on the diamond surface of the single-reflection diamond attenuated total reflectance (ATR) accessory of a Nicolet iS50 FT-IR spectrometer from Thermo Fisher Scientific (Waltham, MA, USA) equipped with a DTGS detector. Typically, for a single ATR FT-IR spectrum, 32 interferograms of 2 cm^–1^ nominal resolution were coadded. Due to the difficulty in determining the real values of refractive indexes of amyloid aggregates, only uncorrected ATR FT-IR data is shown. Spectral data processing was limited to subtracting the water vapor spectrum using GRAMS software (Thermo Fisher Scientific).

### Circular Dichroism Measurements

For circular dichroism (CD) measurements of fresh aqueous solutions of peptide samples (typically at a 0.21 mg/mL concentration), 1 mm quartz cuvettes were used. All CD spectra corrected for the buffer signal were acquired at room temperature by the accumulation of 5 independent spectra (at a 200 nm/s scanning rate) on a J-815 S spectropolarimeter from Jasco Corp. (Tokyo, Japan).

### Molecular Dynamics Simulations

Molecular dynamics (MD) simulations were carried out using the AMBER 18 GPU implementation.^43,44^ We used the FF15ipq force field^45^ to model aggregated peptides which were solvated with the SPC/E-b model of water molecules.^46^ Modeling and docking of ACC_1–13_K_8_/ ACC_1–13_E_8_ monomers were described previously,^18,41,47^ assuming an in-register parallel β-sheet architecture of the fibrils enforced by the low symmetry of the constituent building blocks. Twenty layers of ACC_1–13_K_8_/ACC_1–13_E_8_ were assembled to create the amyloid core architecture. The aggregate was neutralized with Na^+^/Cl^–^ ions and immersed in a periodic box so that the minimum distance between any peptide atom and the edge of the periodic box became at least 25 Å. The resulting box (83 × 90 × 140 Å^3^) contained around 33,000 water molecules in total. Initially, the system was minimized (5000 steps), gradually heated to 300 K, and finally equilibrated over a 10 ns period using the NPT ensemble with a 2 fs time step. This was followed by a 10 ns equilibration of the NVT ensemble. During these stages, positional restraints were applied to all C_α_ carbon atoms with respect to their initial positions with a spring constant of 1.0 kcal/mol/Å^2^. The production phase consisted of three independent runs, each 500 ns long, for every considered amyloid structure (ACC_1–13_K_8_-ACC_1–13_E_8_, ACC_1–13_K_8_-only, and ACC_1–13_E_8_-only) in the NVT ensemble and without any positional restraints. We also employed the molecular mechanics Poisson Boltzmann surface area (MMPBSA) method to estimate the binding energy of the ACC_1–13_E_8_-ACC_1–13_K_8_ assembly. The details have been placed in the Supporting Information.

## Results and Discussion

**Figure 1:**
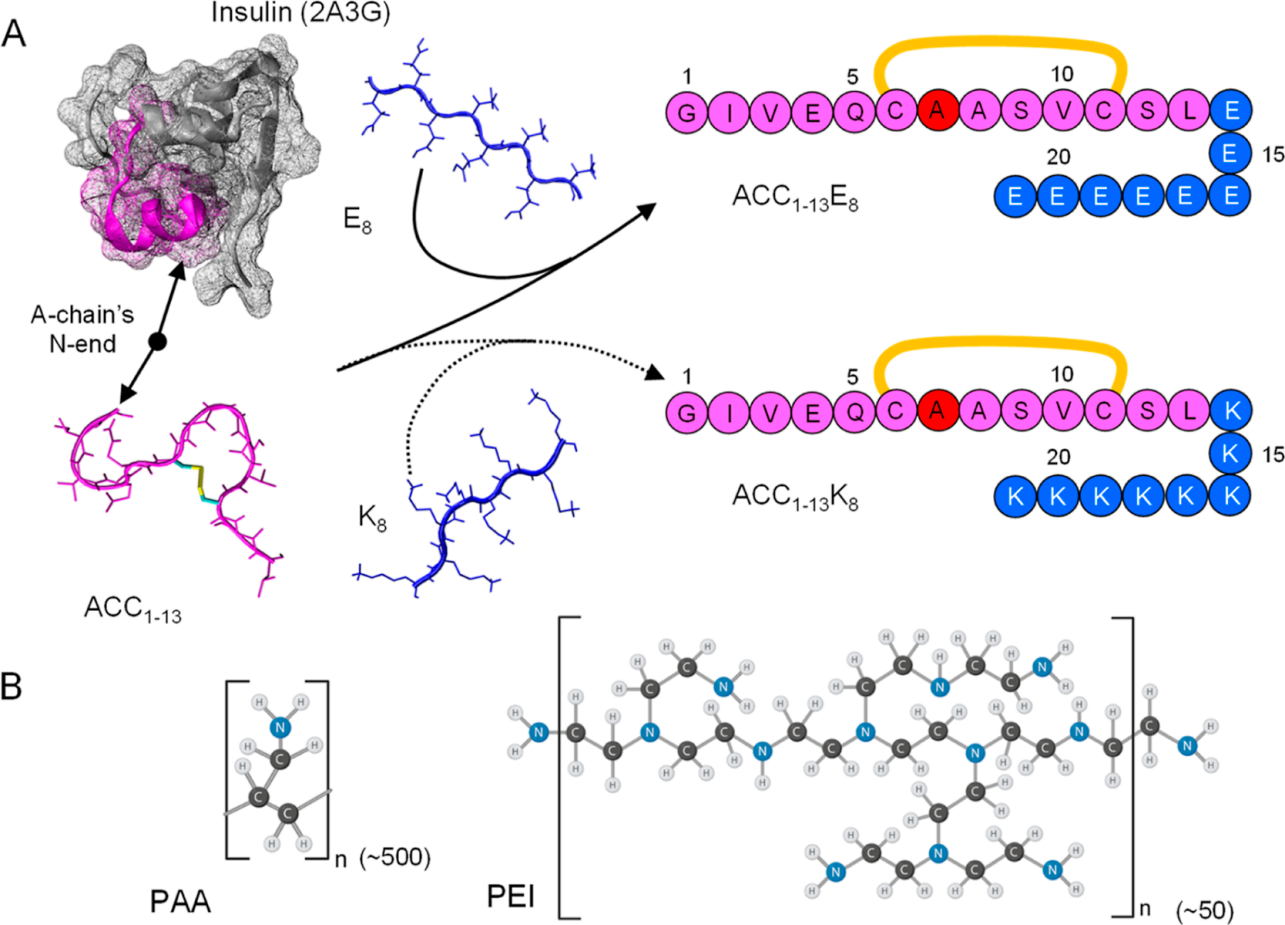
(A) Design of the ACC_1–13_E_8_ and ACC_1–13_K_8_ peptides. The amino acid sequence of the N-terminal segment of bovine insulin’s A-chain (the first 13 residues) was extended at the C-end by additional 8 glutamate or 8 lysine residues. (B) Nonbranched and branched structures of monomer units of PAA and PEI, respectively. Averaged degrees of polymerization are given in parentheses.

Within the structure of a natively folded insulin monomer, the N-terminal disulfide-constrained segment of A-chain (ACC_1–13_) is mostly α-helical, concealing its profoundly amyloidogenic character.^37–39^ The extreme tendency to aggregate and form fibrils is revealed when short ACC_1–13_-containing fragments of insulin are released into the solution upon partial enzymatic proteolysis of the parent protein^37^ or when this segment is engineered into various chimeric peptides (Figure 1).^38–42^

**Figure 2:**
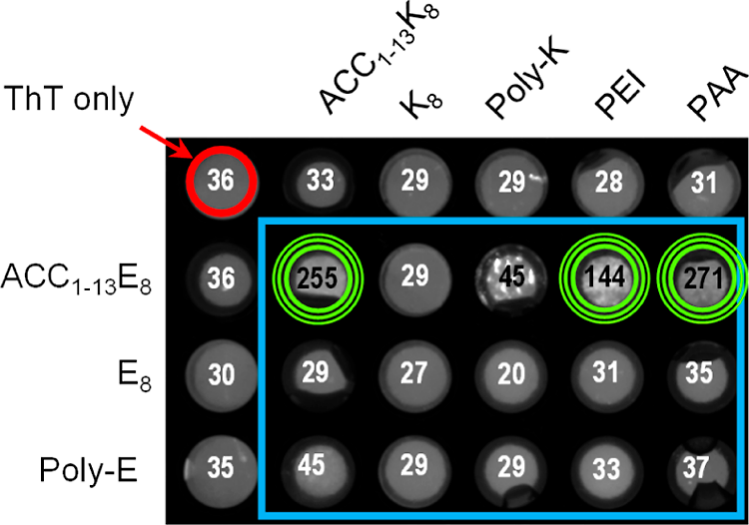
ThT fluorescence-based screening for amyloidal aggregates formed upon the mixing of aqueous solutions of selected oligocations and oligoanions at pH 7 and subsequent incubation. Numerical values superimposed on the UV-illuminated plate image correspond to ThT emission readouts (λ_ex._ 440 nm/λ_em._ 485 nm) collected for wells filled with nonmixed (top row and far left column) and mixed (within the blue frame) solutions of specified compounds after 48 h of incubation at 37 °C. The final concentration of ACC_1–13_E_8_ was 0.5 mg/mL, while the concentrations of added counterions were calculated assuming a 1:1 charge compensation stoichiometry and full ionization of all carboxyl and amine (primary and secondary) groups. Each well contained ThT at a 30 μM concentration. The most fluorescing samples are indicated with green rings; the control readout for the neat ThT solution is marked with a red ring.

We have shown previously that coupling of ACC_1–13_ with octalysine^41^ or octaglutamate^42^ produces peptides (ACC_1–13_K_8_ and ACC_1–13_E_8_, respectively), which on their own do not aggregate at neutral pH due to repulsive interactions between large electric charges on the monomers. Coupling of extremely amyloidogenic protein fragments with segments bearing large uncompensated electric charges creates frustrated peptide units whose rapid fibrillization at a close-to-neutral pH is conditioned on the presence of compatible counterions. Apart from the charge itself, several different factors (e.g., size, structural flexibility, and periodicity in the spatial distribution of charges) are expected to determine whether a particular counterion could become a competent match for the frustrated peptide to coassemble into fibrils: one of the key problems in, for example, amyloidogenesis of Tau protein.^48^ We began this study by inquiring how promiscuous ACC_1–13_K_8_ and ACC_1–13_E_8_ are in selecting the charge-compensating partner by designing a series of paired (in terms of electrostatics) coassembly systems progressing from a potentially perfect structural match (ACC_1–13_K_8_ and ACC_1–13_E_8_ coassembly) to a profound mismatch exemplified by ACC_1–13_E_8_ interacting with nonpeptidic linear and branched polyamines such as PAA and PEI (Figure 1B). In this series, we have also included separate K_8_ and E_8_ fragments and long poly-K and poly-E homopolypeptides. The preliminary screening test was carried out at neutral pH and at the theoretically optimal mixing stoichiometry, i.e., at a 1:1 ratio of negative/positive electric charges on mixed components while assuming that at pH 7, (i) all carboxyl groups in ACC_1–13_E_8_, E_8_, and poly-E are ionized and (ii) all amine groups in ACC_1–13_K_8_, K_8_ poly-K, PAA, and PEI are protonated. Neat aqueous solutions of all these polyanions and polycations containing added ThT were mixed pairwise in wells of a standard 96-well plate (or in Eppendorf tubes) and subjected to a 48 h-long incubation at 37 °C. In Figure 2, a monochromatic image of the plate illuminated with ThT-fluorescence-exciting UV light is presented along with the numerical values of ThT emission intensity collected for all wells. One result immediately clear from the screening test is that coaggregations of ACC_1–13_E_8_-ACC_1–13_K_8_, ACC_1–13_E_8_-PAA, and ACC_1–13_E_8_-PEI result in ThT-positive products.

**Figure 3:**
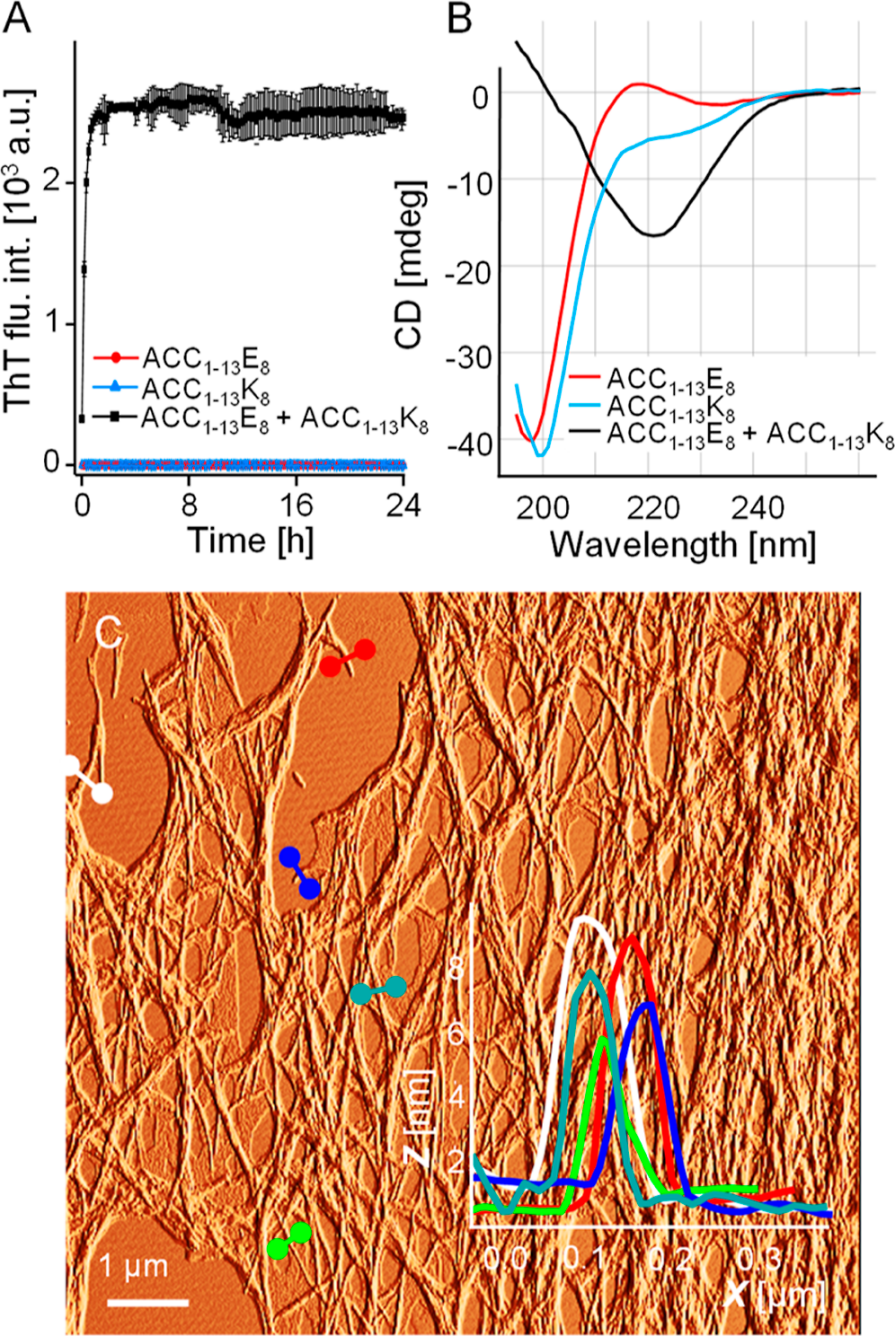
Cofibrillization of ACC_1–13_E_8_ and ACC_1–13_K_8_. (A) ThT fluorescence-based monitoring of fibrillization of 0.22 mM aqueous solutions of ACC_1–13_E_8_, ACC_1–13_K_8_, and their equimolar mixture; pH 7, 20 μM ThT, 37 °C, 300 rpm, 24 h. (B) Far-UV CD spectra of aqueous suspension of ACC_1–13_E_8_-ACC_1–13_K_8_ coaggregate juxtaposed with the spectra of individual peptides at pH 7 (0.11 mM conc., 1 mm optical pathway). (C) Amplitude AFM image of ACC_1–13_E_8_-ACC_1–13_K_8_ coaggregate; overlaid are cross sections of the selected fibrillar specimen.

Interestingly, no similarly enhanced ThT fluorescence was observed for the pairs of ACC_1–13_E_8_-K_8_, ACC_1–13_K_8_-E_8_, ACC_1–13_K_8_-poly-E, and ACC_1–13_E_8_-poly-K (in the last two cases, the minor increase of fluorescence readout from the range 25–38 to approximately 45 au is negligible in comparison to the three most fluorescing samples). The lack of ThT emission enhancement in the poly-E-poly-K sample is not surprising since poly-l-lysine is known to coaggregate with poly-l-glutamic acid to form an amorphous nonfibrillar aggregate based on the motif of antiparallel β-sheet.^47^ Following the outcome of this initial high-throughput screening, we have focused on the processes (and their respective products) occurring when dissolved ACC_1–13_E_8_ interacts with ACC_1–13_K_8_, PAA, or PEI. The trajectories presented in Figure 3A correspond to time-dependent changes in ThT intensity observed upon rapid mixing of stoichiometric portions of ACC_1–13_E_8_ and ACC_1–13_K_8_ solutions at neutral pH. The rapid gain in signal intensity contrasts with the flat trajectories collected for the nonmixed peptide samples.

**Figure 4:**
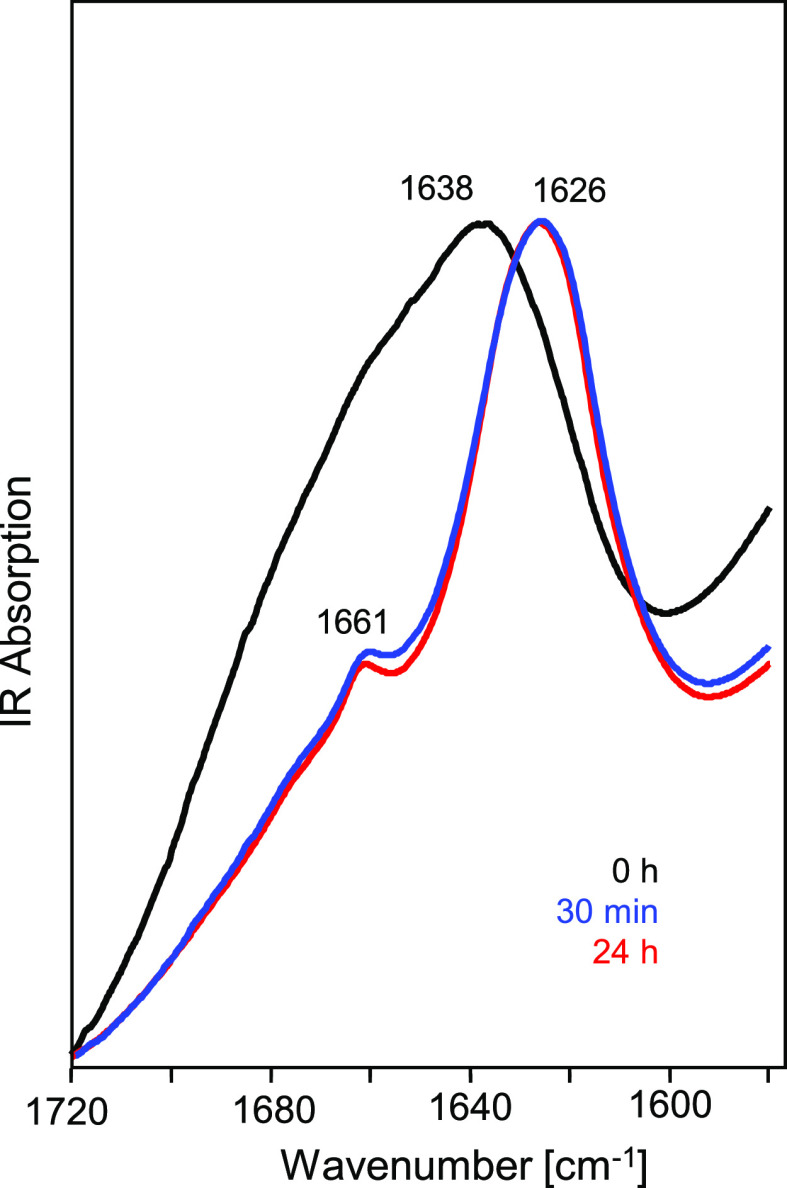
Time-lapse ATR FT-IR spectra (amide I band region) of an equimolar mixture of ACC_1–13_E_8_ and ACC_1–13_K_8_, pH 7, undergoing spontaneous coaggregation while incubated in a thermoblock at 37 °C.

**Figure 5:**
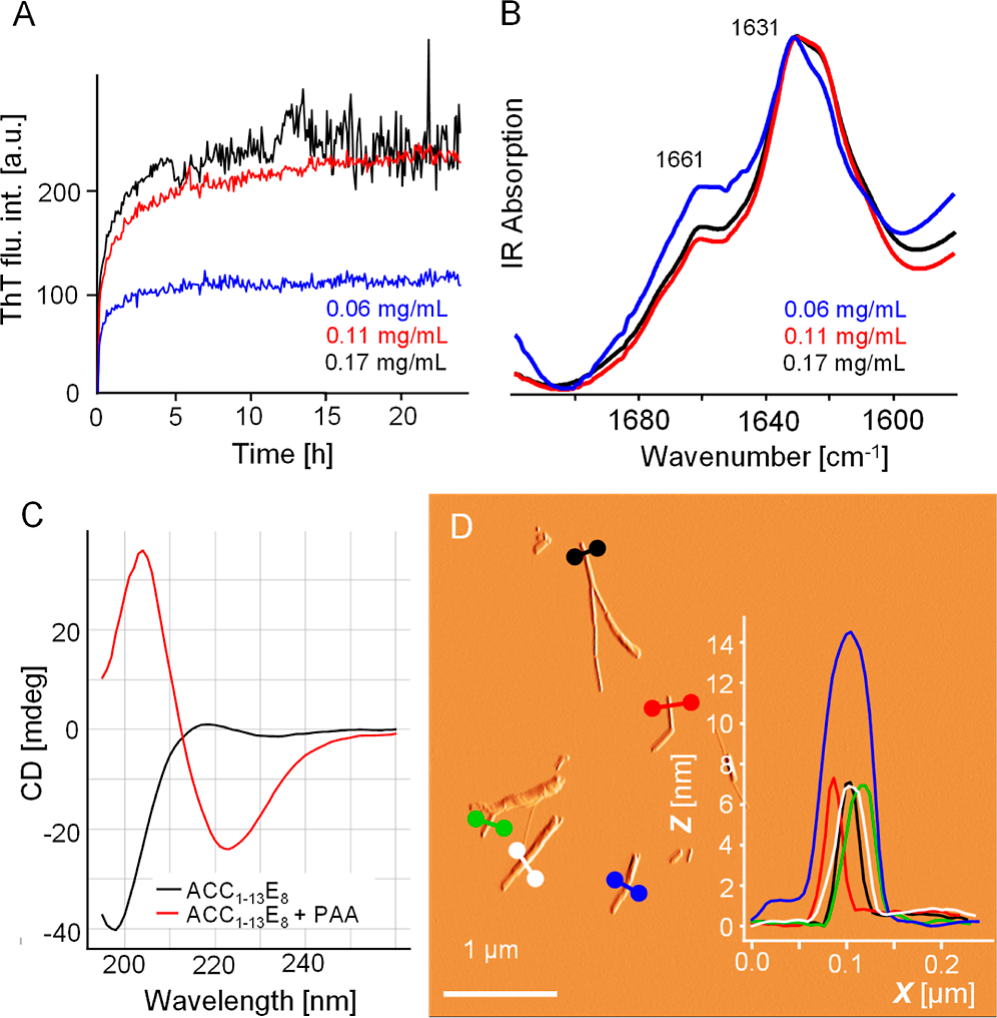
Coaggregation of ACC_1–13_E_8_ and PAA. (A) ThT fluorescence trajectories obtained for samples containing ACC_1–13_E_8_ at fixed concentrations of 0.22 mM and various concentrations of PAA, as indicated. Other conditions of fibrillization: pH 7, 30 μM ThT, 37 °C, 300 rpm, 24 h. (B) FT-IR spectra of dry coaggregates collected afterward. (C) Far-UV CD spectra of aqueous suspension of ACC_1–13_E_8_-PAA coaggregate and neat ACC_1–13_E_8_ both at pH 7 (0.11 peptide mM conc., 1 mm optical pathway). (D) Amplitude AFM image of ACC_1–13_E_8_-PAA coaggregates; overlaid are cross sections of the selected fibrillar specimen.

**Figure 6:**
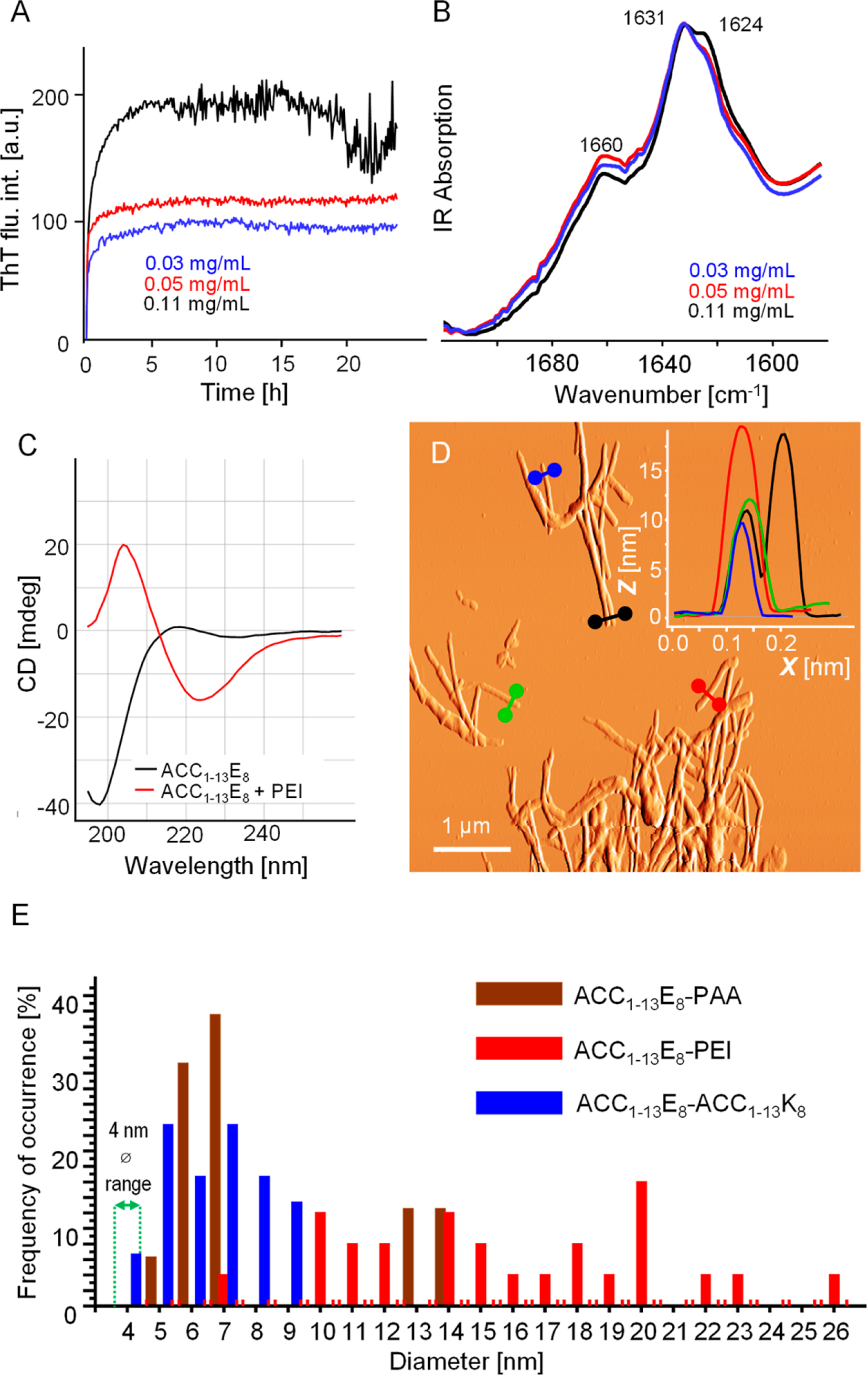
Coaggregation of ACC_1–13_E_8_ and PEI; comparison of the thickness of various aggregates. (A) ThT fluorescence trajectories obtained for samples containing fixed concentrations of ACC_1–13_E_8_ (0.22 mM) and various indicated concentrations of PEI. Other conditions of fibrillization: pH 7, 30 μM ThT, 37 °C, 300 rpm, 24 h. (B) FT-IR spectra of dry coaggregates collected afterward. (C) Far-UV CD spectra of aqueous suspension of ACC_1–13_E_8_-PEI coaggregate and neat ACC_1–13_E_8_ both at pH 7 (0.11 peptide mM conc., 1 mm optical pathway). (D) Amplitude AFM image of ACC_1–13_E_8_-PEI coaggregate; overlaid are cross sections of the selected fibrillar specimen. (E) Histogram representation of relative abundancies of fibrillar specimens of various widths (estimated according to the AFM height images) in aggregates of ACC_1–13_E_8_-ACC_1–13_K_8_, ACC_1–13_E_8_-PAA, and ACC_1–13_E_8_-PEI.

The lack of a detectable lag phase and the very steep increase in ThT emission up to the final plateau remind us of the fibrillization behavior of other single-component ACC_1–13_-derived systems (e.g., ref (39)). We have confirmed that the “explosive” formation of the ThT-positive precipitate coincides with a transition on the level of the secondary structure. The far-UV-CD spectra in Figure 3B indicate that, separately, both the peptides are disordered in neutral-pH aqueous solutions, but the coaggregate is composed of the β-sheet structure reflected by the single minimum slightly above 220 nm (the shift from the 216 nm minimum is commonly observed for amyloid fibrils^39^). The most tangible proof of the amyloidal character of ACC_1–13_E_8_-ACC_1–13_K_8_ coassemblies was obtained through the application of AFM. The amplitude image in Figure 3C reveals plenty of laterally aligned fibrils in the sample collected at the end of the kinetic experiment reported in Figure 3A. The thinnest individual specimens are 6–8 nm in diameter, a rather large value for a single protofilament composed of peptides of this size, suggesting that these are already higher-order structures composed of several intertwined protofilaments. The appearance of these fibrils is otherwise typical for amyloid aggregates with strong tendencies to form superstructures as is both the case of insulin^49^ and, for example, ACC_1–13_K_8_ incorporating ATP.^41^ As a complementary tool to probe the secondary structure of aggregates, infrared spectroscopy was employed. In Figure 4, time-lapse infrared spectra of the ACC_1–13_-ACC_1–13_K_8_ system in the conformation-sensitive amide I band region are shown. The broad spectral contour of the band with the maximum at ca. 1638 cm^–1^ corresponds to the superimposition of the signals of individual yet disordered component peptides. The red shift of the band to 1626 cm^–1^, its pronounced narrowing, and the absence of the high-frequency exciton-split component above 1680 cm^–1^ are all indicative of the parallel β-sheet structure. The minor spectral component at 1660 cm^–1^ is likely to arise from turns. The dramatic evolution of the infrared spectra appears to be complete within the 30 min of coincubation of ACC_1–13_E_8_ and ACC_1–13_K_8_, as the spectra collected after 24 h are practically the same. We have followed essentially the same protocol to verify the amyloidal characters of the coaggregates formed by the two more puzzling pairs: ACC_1–13_E_8_-PAA (Figure 5) and ACC_1–13_E_8_-PEI (Figure 6). When two distinct macromolecular building blocks (different in terms of main-chain lengths, backbone flexibilities, and periodicities with which charged side groups are distributed alongside the main chains) self-assemble, the nominal 1:1 stoichiometry of mixing (quantified by the numbers of charge-bearing groups) may be suboptimal, as steric hindrances could prevent local saturation of pairwise ionic interactions between the two components. Furthermore, the actual p*K*_a_ value of chemically identical ionizable side groups within a macromolecule is unlikely to be uniform (e.g., ref (50)) and will ultimately depend on the local environment—e.g., polarity and presence of counterions. For this reason, we tested ThT-fluorescence-monitored fibrillization at slightly altered mixing ratios of the two components (Figures 5A and 6A).

In the case of the ACC_1–13_E_8_-PAA pair (Figure 5A), decreasing PAA concentration to the level corresponding to an approximately 1:3 molar ratio of amine (from PAA) groups/glutamate (from ACC_1–13_E_8_) groups results in a lower final plateau of ThT intensity. Hence, the concentration of the counterion clearly controls the amount of amyloid formed. We note that a more qualitative utilization of the ThT intensity for “titration” experiments would be problematic since ThT is cationic, and thus repulsive interactions between the fluorophore and protonated polyamine chains could kick in, causing nonlinear deviations in the relationship between the concentration of amyloid saturated with PAA (to various degrees) and the ThT signal.

For all three PAA concentrations examined, the ThT-fluorescence-monitored transition is very fast without presenting a detectable lag phase. The infrared spectra of coaggregate samples collected at the end of the kinetic experiment (Figure 5B) point to the presence of a predominantly parallel β-sheet structure (amide I band’s maximum is at 1631 cm^–1^). At the two higher PAA concentrations, the low-wavenumber spectral component below 1629 cm^–1^ is slightly elevated. This, however, should not be interpreted as an indication of PAA-controlled polymorphism of fibrils (e.g., appearance of aggregates with weakened interstrand hydrogen bonds). In unison with the ATR FT-IR spectra, far-UV-CD data shown in Figure 5C supports the presence of a β-sheet structure of the ACC_1–13_E_8_-PAA coaggregate. Finally, the fibrillar character of ACC_1–13_E_8_-PAA was confirmed by using AFM (Figure 5D). The fibers turned out to be moderately thick (6–14 nm in diameter), rather short, and singly dispersed. One could speculate that while the incorporation of PAA may locally compensate for negative charges on E_8_ segments, excesses of positively charged polyamine layers could disfavor lateral assembly of fibrils through electrostatic coat-to-coat repulsion. The analogous set of kinetic, infrared, CD, and AFM results obtained for the ACC_1–13_E_8_-PEI pair and presented in Figure 6 reveal a surprising level of similarity in how these two very different polyamines trigger fibrillization of the peptide. Within the repetitive building unit of PEI (Figure 1B), the ratio of primary/secondary/tertiary amine moieties is 4:3:4. All of these amine groups could potentially be protonated at neutral pH, especially in the presence of ionic-pair-forming counterions. The spatial accessibility of these amine groups is different, however. Hence, while considering the effect of PEI concentration on the kinetics of coaggregation with ACC_1–13_E_8_ (Figure 6A), one should take this additional layer of complexity into account, as well. Overall, the branched structure of PEI clearly poses no obstacles in triggering the formation of a highly ordered β-sheet structure (as reflected by the infrared and CD spectra shown in Figure 6B,C). We note that the resulting fibrils tend to be slightly thicker and laterally aligned to a higher degree than those of ACC_1–13_E_8_-PAA (Figure 6D). In fact, this has been confirmed through a larger-scale statistical survey of the morphologies of ACC_1–13_E_8_-ACC_1–13_K_8_, ACC_1–13_E_8_-PAA, and ACC_1–13_E_8_-PEI based on a thorough examination of height AFM images of multiple fibrillar specimens.

**Figure 7:**
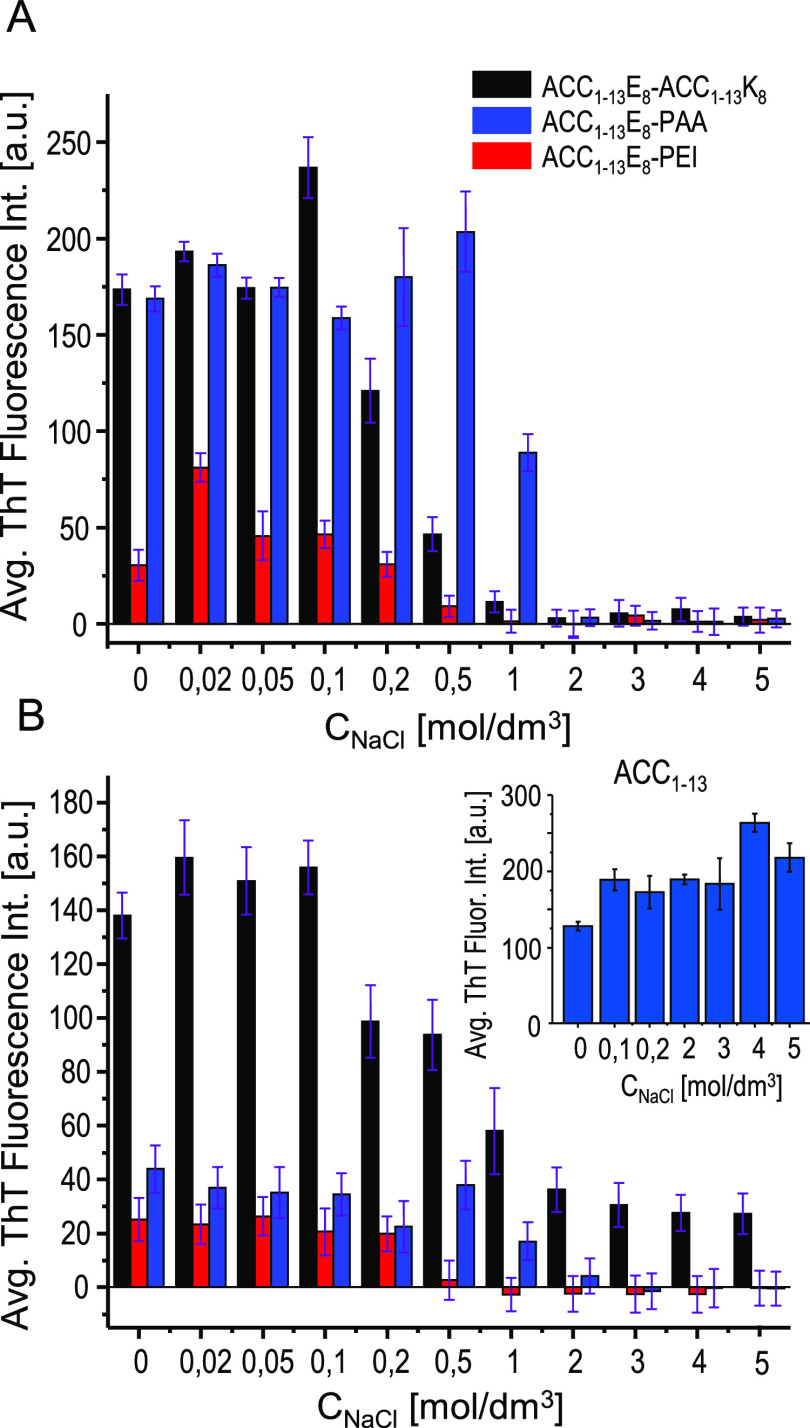
Influence of ionic strength on ACC_1–13_E_8_-polycation cofibrillization and on stability of preformed coaggregates. (A) ThT emission readouts (λ_ex._ 440 nm/λ_em._ 485 nm, 30 μM ThT) averaged over the third hour of incubation at 37 °C of ACC_1–13_E_8_ (0.5 mg/mL) mixed with ACC_1–13_K_8_, PAA, or PEI in the presence of increasing NaCl concentration. The mixing stoichiometry of all negatively and positively ionized groups was 1:1, assuming full ionization of all carboxyl and amine (primary and secondary) groups (the same conditions as in Figure 2), pH 7. (B) ThT emission readouts of ACC_1–13_E_8_-polycation coaggregates formed under typical conditions (1:1 ionic stoichiometry of mixing, pH 7, 24 h incubation at 37 °C, without NaCl) and subsequently transferred to aqueous NaCl solutions of specified concentrations, pH 7, containing 30 μM ThT. The data correspond to emission values averaged over 18 h of incubation at 37 °C. The control data on the NaCl effect on ThT-stained fibrils of ACC_1–13_ incubated under analogous conditions is shown in the inset.^40^

The key result of this analysis, which focused on fibrils’ diameters, has been concisely presented in Figure 6E. It appears that the overall tendency to promote the formation of thick fibers at the expense of thinner forms increases in the order in which the three coaggregates are presented here with ACC_1–13_E_8_-PEI fibrils revealing the particular tendency to form higher-order structures. One may speculate that the polyamine chains (especially with branches) enhance the lateral assembly of fibrils into larger bundles by cross-interacting with ACC_1–13_E_8_ chains involved in separate filaments. We have also attempted to estimate the persistence lengths of all three types of fibrils based on the obtained AFM data (Supporting Information). The outcome of these estimations suggests that the persistence length of ACC_1–13_E_8_-ACC_1–13_K_8_ fibrils (∼2.3 μm) exceeds those of ACC_1–13_E_8_-PAA (∼1 μm) and ACC_1–13_E_8_-PEI (∼1.5 μm), implying that the local structural match of ACC_1–13_E_8_/ACC_1–13_K_8_ units results in an increased overall structural stiffness of fibrils. These results should be treated cautiously, however, since the singly dispersed and mechanically relaxed fibrillar specimens were scarce in the input data. The results presented to date provide compelling evidence that ACC_1–13_E_8_ is capable of forming two-component amyloid fibrils when matched with a competent (in terms of charge complementarity and structure) partner. As Coulombic interactions appear to play an essential role in stabilizing the mixed fibrils, we have examined how increasing ionic strength would affect both the process of de novo coaggregation of ACC_1–13_E_8_ with cationic partners and the stability of coaggregates preformed at negligible ionic strength. Recently, modulation of ionic strength conditions by changing the concentration of added NaCl proved very insightful in a study on the formation of liquid droplets and coaggregation of ACC_1–13_K_*n*_ (*n* = 8, 16, 24, 32, 40) peptides and ATP.^51^ Here, we use the same approach. In Figure 7A, the impact of codissolved NaCl on early gains in ThT emission intensity reflecting an amyloid buildup in mixed ACC_1–13_E_8_-polycation samples is presented. While high salt concentrations (2 M and above) universally prevent coaggregation in all three systems by weakening the Coulombic driving forces, at the low NaCl concentration edge the picture is more nuanced. At 20 mM NaCl, ACC_1–13_E_8_-PEI coaggregation appears to be transiently enhanced, perhaps through decreasing barriers associated with escaping kinetic traps.^51^ However, the same system along with ACC_1–13_E_8_-ACC_1–13_K_8_ coaggregation is quite susceptible to higher salt concentrations (coaggregation practically does not occur above 0.5 M NaCl), whereas ACC_1–13_E_8_-PAA coaggregation is still efficient in the presence of 1 M NaCl. As the data shown in Figure 7B indicates, high ionic strength not only prevents de novo coaggregation but also causes disassembly of the preformed coaggregates (while it has no impact on ACC_1–13_ fibrils whose self-assembly does not rely on Coulombic interactions; see the control data in the inset there). The added NaCl appears to have less of an impact on preformed ACC_1–13_E_8_-ACC_1–13_K_8_ fibrils than on the process leading to their formation. This hysteresis-like behavior may be interpreted as an indication that early stages of ACC_1–13_E_8_-ACC_1–13_K_8_ coaggregation involve intermediates or phases (such as liquid droplets) stabilized by Coulombic forces and therefore are particularly vulnerable to high salt concentrations.

**Figure 8:**
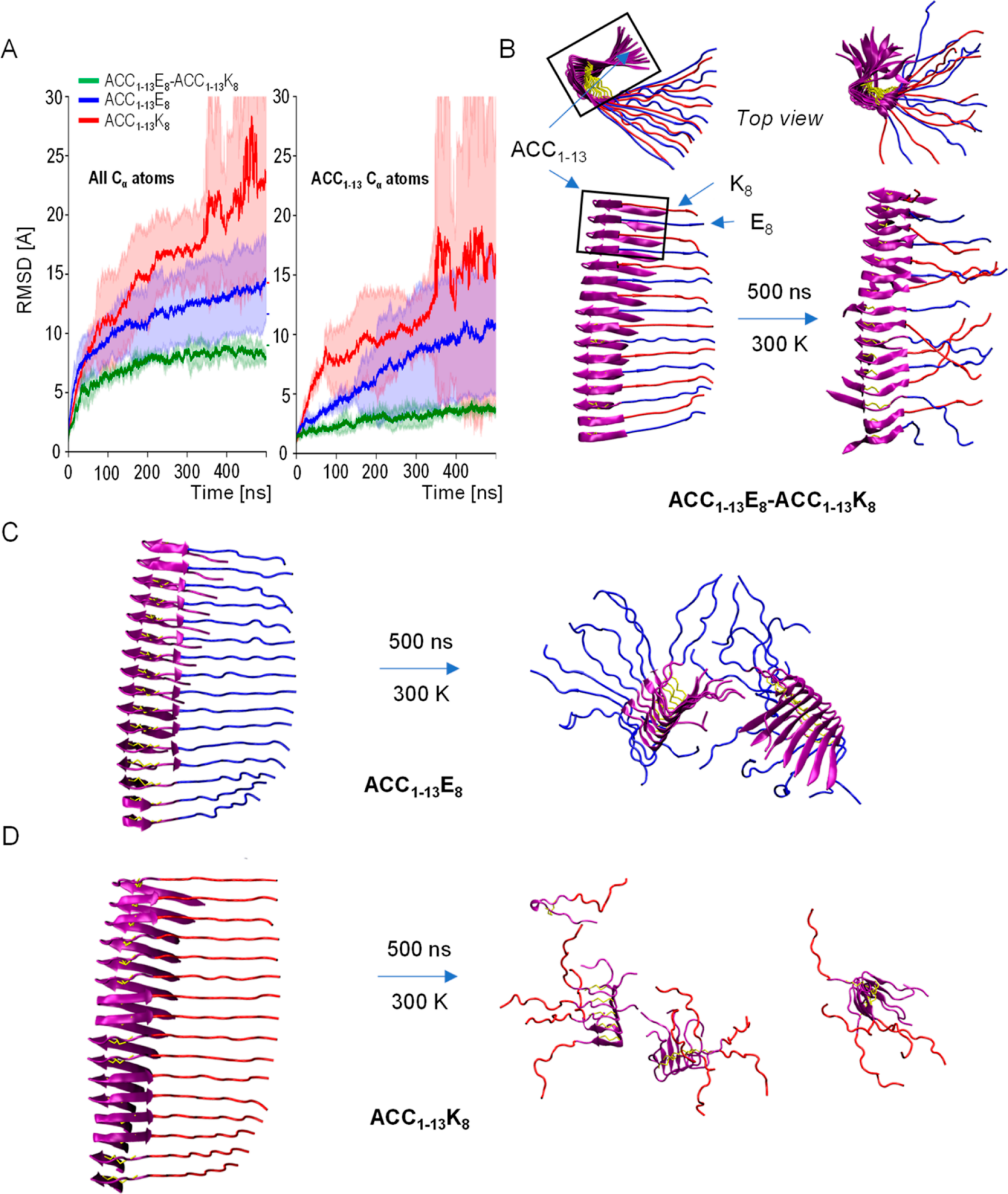
MD-based analysis of stability of ACC_1–13_E_8_-ACC_1–13_K_8_ coaggregate. (A) Time-dependent changes of RMSD of C_α_ atoms at 300 K in preassembled fibrillar stacks of extended peptides chains (in-register parallel β-sheet structure): alternate layers of ACC_1–13_E_8_ and ACC_1–13_K_8_ versus analogous structures composed of ACC_1–13_E_8_ and ACC_1–13_K_8_ only. The presented averaged RMSD trajectories have been calculated for all C_α_ atoms (left) and C_α_ atoms within the ACC_1–13_ segments only (right). (B–D) Snapshots of the initial and final (after 500 ns) states of these structures.

The fact that stoichiometric amounts of ACC_1–13_E_8_ and ACC_1–13_K_8_ self-assemble into amyloid fibrils is perhaps the most intuitive. For low-symmetry building blocks such as disulfide-bonded ACC_1–13_*X*_*n*_ peptides, one efficient way to form aggregates with saturated interstrand hydrogen bonds and van der Waals interaction is to align neighboring monomers in the motif of an in-register parallel β-sheet structure.^18^ Individual peptide chains are in a quasi-translational relationship within the resulting linear aggregate, which bears all the structural hallmarks of an amyloid protofilament. This symmetry-based argument has been used earlier to visualize a plausible structure of the ACC_1–13_K_8_-ATP amyloid.^41^ The same approach has been employed here to build a structural model of an ACC_1–13_E_8_-ACC_1–13_K_8_ amyloid aggregate: once alternating monomers of both peptides were preassembled in planarized extended conformations, the structural constraints were removed and all-atom MD simulations in an explicit solvent followed. The results are presented in Figure 8.

RMSD trajectories obtained for 500 ns-long simulations point to, rather predictably, a gain in stability of the mixed ACC_1–13_E_8_-ACC_1–13_K_8_ assemblies vis-à-vis assemblies composed of a single type of peptide in the same initial arrangement with the electric charges on the polypeptide chains being compensated by the added Na^+^ or Cl^–^ ions. According to the snapshot of the ACC_1–13_E_8_-ACC_1–13_K_8_ structure after 500 ns of simulation, the ACC_1–13_ segment preserves the β-sheet conformation to a larger degree than the sheets of alternating K_8_-E_8_ strands do. It is important to note that in a simulation of a single protofilament (a limitation due to computational costs), charged lysine and glutamate side chains may form ionic pairs only with partners from the same sheet, which, due to local strains, could potentially compromise the stability of single K_8_-E_8_ sheets (in intertwined protofilaments, ionic interactions could form between side chains across different sheets). It is unclear to what extent the relative destabilization of these segments, visible in the MD simulation, should be attributed to this factor. Importantly, one should stress that according to the infrared data shown earlier (Figure 4), ACC_1–13_E_8_-ACC_1–13_K_8_ fibrils are highly ordered in terms of the secondary structure. The dominant spectral component assigned to the parallel β-sheet structure is consistent with K_8_-E_8_ segments becoming β-sheet-like, as well. Because of the inherent methodological limitations of MD, the content and stability of the extended structure in the mixed aggregate may be underestimated, yet the demonstration of interactions between ACC_1–13_ segments being crucial contributors to the fibrils’ stability is sound. Of note: the RMSD levels calculated for C_α_ atoms within the ACC_1–13_ segments of mixed ACC_1–13_E_8_-ACC_1–13_K_8_ fibrils are consistently below the values obtained when all C_α_ atoms are taken into account (Figure 8A). The preliminary screening experiment (Figure 2) has shown that interactions of ACC_1–13_E_8_ with K_8_ only (and likewise ACC_1–13_K_8_ with E_8_) do not produce fibrils. Hence, in our system, the possibility of saturation of Coulombic interactions between strands is an insufficient driving force of the aggregation when the system is locally frustrated at the structural voids between hydrophobic fragments of insulin. We have also used the MMPBSA approach to estimate the binding energy within the ACC_1–13_E_8_-ACC_1–13_K_8_ coassembly.

While obtaining sound results using such computational tools is often rather challenging,^52,53^ it should be noted that negative enthalpy changes compensate for unfavorable entropic cost in the coassembly consisting of at least three ACC_1–13_E_8_-ACC_1–13_K_8_ layers (Supporting Information).

One of the most surprising findings of this study is the capacity of the two nonpeptidic polyamines to coassemble with ACC_1–13_E_8_ while polylysine, with the periodicity of positively charged side chains potentially matching that of the octaglutamate stretch, does not act in a similar way (as is also the case of poly-E and ACC_1–13_K_8_). This is particularly striking in the cases of ACC_1–13_E_8_ and PEI, given the branched character of this polyamine. Clearly, the conformational flexibility of these polycations takes precedence over the similarities in charge periodicity and covalent structure of the backbone of ACC_1–13_E_8_. This level of structural promiscuity in adapting a charge-compensating partner reminds one of Tau protein which (when non-hyperphosphorylated) coaggregates with seemingly incompatible polyanions including heparin, RNA, poly-E, or anionic micelles.^48^ Also, in parallel to the case of Tau coaggregates, PAA and PEI are very unlikely to partition into an amyloid core built of densely packed ACC_1–13_ layers. Instead, the polycations are likely to interact with E_8_ chains by wrapping them with a positively charged fuzzy coat. Due to a number of technical issues and computational costs, in silico modeling of ACC_1–13_E_8_-PAA and ACC_1–13_E_8_-PEI aggregates proved to be too challenging at this point. There are several aspects of the puzzling ability of amyloid architectures to adapt to perturbations which seem to be entirely inconsistent with these highly ordered structures. For example, it was demonstrated that copolymer poly-α-amino acids with randomized amino acid sequences can form amyloid fibrils.^54^ Foreign components of amyloid fibrils such as heparin, RNA, or ATP may assist in the formation of abnormal protein aggregates without partitioning into the amyloid core. Such macromolecules may stabilize fibrils by offsetting undesirable interactions within structurally dynamic external “fuzzy coat” layers, as is the case of Tau coaggregating with poly-E or ACC_1–13_E_8_-PAA/-PEI. These nonproteinaceous agents may also impact earlier stages of protein misfolding—for example, by assisting liquid–liquid phase separation, which is now considered to be intimately involved in the etiology of many amyloid-associated diseases.^55–57^

## Conclusions

In conclusion, we have demonstrated that ACC_1–13_E_8_ and ACC_1–13_K_8_, a pair of chimeric peptides designed by coupling the potent amyloidogenic fragment of insulin with octaglutamate/octalysine segments, are robust building blocks for the rapid coassembly of mixed amyloid fibrils. The low symmetry of bent conformers of these peptides (due to the intact Cys7–Cys11 disulfide bridge) constitutes a strong argument that saturation of interstrand hydrogen bonds, van der Waals interactions, and salt bridges between glutamate and lysine side chains would be achieved through a self-assembly mode consistent with an in-register parallel β-sheet. The infrared and MD data resonate with this idea. The role of Coulombic interactions in the amyloidogenic coassembly of ACC_1–13_E_8_ with ACC_1–13_K_8_, PAA, and PEI is reflected by the sensitivity of these processes and mixed fibrils to high ionic strength conditions. Highly flexible chains of two polyamines, PAA and PEI, turned out to be more effective in triggering amyloid fibrils from ACC_1–13_E_8_ than poly-K, suggesting that conformational elasticity is an essential selection criterion for competent partners for the self-assembly of mixed fibrils. We argue that the presented two-component systems are insightful mechanistic models to study the mechanisms of fibrillization of proteins involving formation of fuzzy coats.

## Data Availability

The data are available from the authors on reasonable request.

## Supporting Information Available

Estimation of the persistence length of fibrils and MMPBSA calculations of ACC_1–13_E_8_-ACC_1–13_K_8_ assembly (PDF)
