# Supplementary material for: Self-Assembly of Insulin-Derived Chimeric Peptides into Two-Component Amyloid Fibrils: The Role of Coulombic Interactions
Source: J Phys Chem B. 2023 Jul 26;127(30):6597–607. doi: 10.1021/acs.jpcb.3c00976 (PMC10405213; doi:10.1021/acs.jpcb.3c00976)
Supplement: Supplementary file 1 — jp3c00976_si_001.pdf [file jp3c00976_si_001.pdf]

Self-assembly of insulin-derived chimeric peptides into two-component amyloid fibrils: the role of Coulombic interactions

Mateusz Fortunka, Robert Dec, Wojciech Puławski, Marcin Guza, and Wojciech Dzwolak

Supporting Information

1. ***Estimation of the persistence length of fibrils***
2. ***MMPBSA (Molecular Mechanics – Poisson-Boltzmann Surface Area) calculations***

1. *Estimation of the persistence length of fibrils*

We estimated the persistence length, *p,* using formula

$$\langle E^2\rangle_{2D} = 4pc[1 - 2(1 - e^{-c/2p})p/c],$$

where *E* is an end-to-end distance on a two-dimensional surface (2D) of the fibril and c denotes its contour length [1]. *E* and *c* were traced measured using SNT module in Fiji software [2,3]. The input data were taken from AFM images collected in this work.

Fibril type	Persistence length
$ACC_{1\text{-}13}E_8$-$ACC_{1\text{-}13}K_8$ (n = 15)(1)	2.31 ± 0.27 µm (2)
$ACC_{1\text{-}13}E_8$-PAA (n = 6)	1.04 ± 0.08 µm
$ACC_{1\text{-}13}E_8$-PEI (n = 14)	1.50 ± 0.15 µm

(1) number of data points
(2) standard deviation errors of the parameter's estimation

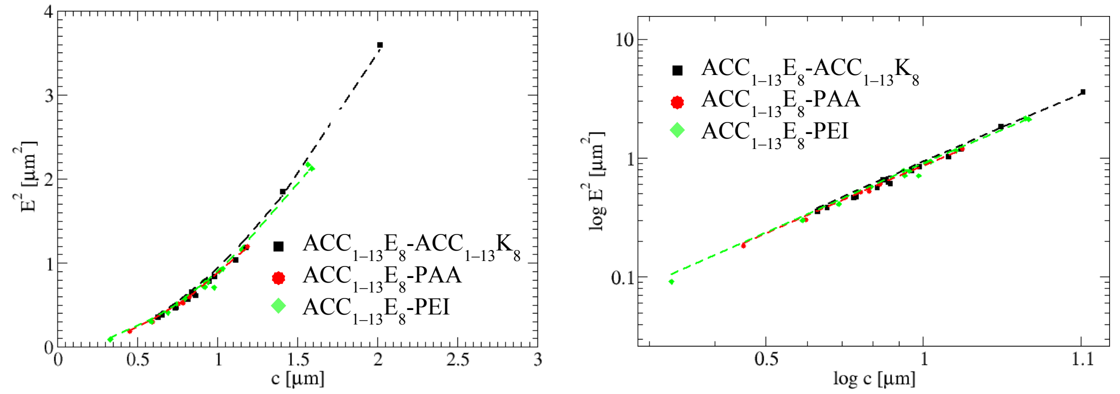

Figure. SI 1 Fits of the squared end to end distance and fibril's contour length.

The graph in Fig. SI 1 presents the fitting results of fibril's end-to-end distance as a function of theirs contour length for the three peptide systems. The solid lines in respective colors represent the best-fit curves obtained for each system.

These estimations of the persistence length must be approached cautiously. The fibrillar species probed by AFM in this study tend to be agglomerated, hence they, as individual specimens, may often not reach the state of mechanical relaxation. The calculated values appear to suggest that the $ACC_{1\text{-}13}E_8$-$ACC_{1\text{-}13}K_8$ fibrils are distinct in terms of structural stiffness among all three co-aggregates.

2. MMPBSA (Molecular Mechanics - Poisson-Boltzmann Surface Area) calculations

We have employed MMPBSA (Molecular Mechanics Poissson Boltzmann Surface Area) method to estimate the binding energy of the alternate $ACC_{1\text{-}13}E_8$-$ACC_{1\text{-}13}K_8$ assembly. The binding free energy, ΔG, between a single layer of $ACC_{1\text{-}13}E_8$ (E) and a consecutive layer of $ACC_{1\text{-}13}K_8$ (K) is calculated as:

$$\Delta G_{\mathrm{bind}} = G_{\mathrm{EK}} - G_{\mathrm{E}} - G_{K},$$

i.e., as the difference between the respective free energies of the complex, G_{EK}, and its components, G_{E}, G_K. The MMPBSA approximates these terms with molecular mechanics (force field) energy, $E_{\mathrm{MM},}$ and implicit solvation effects, G_{sol}:

$$G \sim E_{\mathrm{MM}} + G_{\mathrm{sol}} - TS_{\mathrm{MM}},$$

where T denotes temperature and S_{MM} is an estimation of the entropy.

To this end, we have employed MMPBSA.py script from AmberTools22 [4-5] with the default parameters. We have carried out calculations for a pair of adjacent layers, e.g. layer no. 3 and 4, serving as the 'complex', layer no. 3 served as a 'receptor' and layer no. 4 served as a 'ligand'. In the following step, the two subsequent pairs were considered and an average value is computed. As the symmetry of the fibril imposes periodic effects, we also employ similar scheme for 4 layers, 6, etc. until 16. The two outermost layers in the 20 layer assembly (no. 1, 2, 19, 20) were discarded from computations because of plausible boundary effects. 1500 evenly spaced snapshots were extracted from the second half (3 x 250 – 500 ns) of the MD simulations (see the main article) and they were further used to conduct computations.

Entropy calculations carried out with normal mode approximation converge slowly and are very demanding in terms of computational time [6-7]. Therefore, we report here entropies computed using the quasi-harmonic estimation [8]. For the two layers of $ACC_{1\text{-}13}E_8$-$ACC_{1\text{-}13}K_8$, the estimated entropy of binding is such that $T\Delta S_2$ = -96.90 ± 8.76 kcal/mol. We assume that entropy loss associated with binding of four or more alternate layers can be approximated by multiplying the ΔS_2 by the appropriate number of pairs, n:

$$\Delta S_{Total} \sim n\Delta S_2.$$

The free energy of binding, $\Delta G = \Delta H - T\Delta S$, *can be* normalized *per* the number of pairs:

$$\Delta G/n = (\Delta H - T\Delta S)/n \sim \Delta H/n - T\Delta S_2.$$

where ΔH is the enthalpy calculated for 2 or more pairs.

Table S1. ΔH_{bind} values for $ACC_{1-13}E_8$-$ACC_{1-13}K_8$ aggregate (two layers only).

Time interval [ns]	0.6	3	15	30
ΔH_{bind} [kcal/mol]	-47.00 ± 15.24	-48.06 ± 14.25	-48.40 ± 14.05	-48.16 ± 13.6

Table S2. ΔG / number pairs values for $ACC_{1-13}E_8$-$ACC_{1-13}K_8$ aggregate.

No. of layers dt=15 ns	2	4	6	8	10	12	14	16
ΔH_{bind}/pair [kcal/mol]	-48.40 ± 14.05	-82.45 ± 14.91	-98.59 ± 10.72	-120.97 ± 11.5	-129.42 ± 7.54	-140.53 ± 8.24	-151.93 ± 8.47	-161.01± 7.66
ΔG [kcal/mol]	48.5 ± 16.56	14.45 ± 17.29	-1.69 ± 13.85	-24.07 ± 14.46	-32.52 ± 11.56	-43.63 ± 12.03	-55.03 ± 12.19	-64.11± 11.64

We note that according to these calculations the net thermodynamic stability of $ACC_{1-13}E_8$-$ACC_{1-13}K_8$ aggregate is reached for at least three $ACC_{1-13}E_8$-$ACC_{1-13}K_8$ co-assembling pairs.

References

[1] Rivetti, C., Guthold, M., & Bustamante, C. (1996). Scanning force microscopy of DNA deposited onto mica: EquilibrationversusKinetic trapping studied by statistical polymer chain analysis. ***J. Mol. Biol.*** 264, 919-932.

[2] Arshadi, C., Günther, U., Eddison, M., Harrington, K. I., & Ferreira, T. A. (2021). SNT: a unifying toolbox for quantification of neuronal anatomy. ***Nat. Methods*** *18*, 374-377.

[3] Schindelin, J., Arganda-Carreras, I., Frise, E., Kaynig, V., Longair, M., Pietzsch, T., ... & Cardona, A. (2012). Fiji: an open-source platform for biological-image analysis. ***Nat. Methods*** 9, 676-682.

[4]. D.A. Case, H.M. Aktulga, K. Belfon, I.Y. Ben-Shalom, J.T. Berryman, S.R. Brozell, D.S. Cerutti, T.E. Cheatham, III, G.A. Cisneros, V.W.D. Cruzeiro, T.A. Darden, R.E. Duke, G. Giambasu, M.K. Gilson, H. Gohlke, A.W. Goetz, R. Harris, S. Izadi, S.A. Izmailov, K. Kasavajhala, M.C. Kaymak, E. King, A. Kovalenko, T. Kurtzman, T.S. Lee, S. LeGrand, P. Li, C. Lin, J. Liu, T. Luchko, R. Luo, M. Machado, V. Man, M. Manathunga, K.M. Merz, Y. Miao, O. Mikhailovskii, G. Monard, H. Nguyen, K.A. O'Hearn, A. Onufriev, F. Pan, S. Pantano, R. Qi, A. Rahnamoun, D.R. Roe, A. Roitberg, C. Sagui, S. Schott-Verdugo, A. Shajan, J. Shen, C.L. Simmerling, N.R. Skrynnikov, J. Smith, J. Swails, R.C. Walker, J. Wang, J. Wang, H. Wei, R.M. Wolf, X. Wu, Y. Xiong, Y. Xue, D.M. York, S. Zhao, and P.A. Kollman (2022), Amber 2022, University of California, San Francisco

[5] Miller III, B. R., McGee Jr, T. D., Swails, J. M., Homeyer, N., Gohlke, H., & Roitberg, A. E. (2012). MMPBSA. py: an efficient program for end-state free energy calculations. ***J. Chem. Theory Comput.*** *8*, 3314-3321.

[6] Hou, T., Wang, J., Li, Y., & Wang, W. (2011). Assessing the performance of the MM/PBSA and MM/GBSA methods. 1. The accuracy of binding free energy calculations based on molecular dynamics simulations. ***J. Chem. Inf. Model.*** *51*, 69-82.

[7] Su, P. C., Tsai, C. C., Mehboob, S., Hevener, K. E., & Johnson, M. E. (2015). Comparison of radii sets, entropy, QM methods, and sampling on MM-PBSA, MM-GBSA, and QM/MM-GBSA ligand binding energies of F. tularensis enoyl-ACP reductase (F abI). ***J. Comput. Chem.*** *36*, 1859-1873.

[8] Karplus, M., & Kushick, J. N. (1981). Method for estimating the configurational entropy of macromolecules. ***Macromolecules*** *14*, 325-332.
